# Combined *Ex Vivo* 9.4T MRI and Quantitative Histopathological Study in Normal and Pathological Neocortical Resections in Focal Epilepsy

**DOI:** 10.1111/bpa.12298

**Published:** 2015-09-06

**Authors:** Cheryl Reeves, Mohamed Tachrount, David Thomas, Zuzanna Michalak, Joan Liu, Matthew Ellis, Beate Diehl, Anna Miserocchi, Andrew W. McEvoy, Sofia Eriksson, Tarek Yousry, Maria Thom

**Affiliations:** ^1^ Department of Neuropathology UCL, Institute of Neurology London UK; ^2^ Department of Clinical and Experimental Epilepsy UCL, Institute of Neurology London UK; ^3^ Department of Neuroradiology UCL, Institute of Neurology London UK; ^4^ Department of Brain Repair and Rehabilitation UCL, Institute of Neurology London UK; ^5^ Department of Neurosurgery UCL, Institute of Neurology London UK

**Keywords:** 9.4T MRI, dysplasia, focal epilepsy, quantitative neuropathology

## Abstract

High‐resolution magnetic resonance imaging (MRI) may improve the preoperative diagnosis of focal cortical dysplasia (FCD) in epilepsy. Quantitative 9.4T MRI was carried out (T1, T2, T2* and magnetization transfer ratio) on 13 cortical resections, representing pathologically confirmed FCD (five cases) and normal cortex. Quantitative immunohistochemistry for myelination (myelin basic protein/SMI94), neuronal populations [microtubule‐associated protein 2 (MAP2), neurofilament (SMI31, SMI32), synaptophysin, NeuN, calbindin], reactive glia (GFAP), microglia (CD68) and blood–brain barrier permeability (albumin) was carried out in 43 regions of interest (ROI) from normal and abnormal white matter and cortex. MRI was spatially aligned and quantitative analysis carried out on corresponding ROI. Line profile analysis (LPA) of intensity gradients through the cortex was carried out on MRI and immunostained sections. An inverse correlation was noted between myelin/SMI94 and T1, T2 (*P* < 0.005) and T2* (*P* < 0.05; Spearman's correlation) and a positive correlation between neuronal MAP2 and T1 (*P* < 0.005) and T2* (*P* < 0.05) over all ROI. Similar pathology–MRI correlations were observed for histologically unremarkable white matter ROI only. LPA showed altered gradient contours in regions of FCD, reflecting abnormal cortical lamination and myelo‐architecture, including a preoperatively undetected FCD case. This study demonstrates the ability of quantitative 9.4T MRI to detect subtle differences in neuronal numbers and myelination in histologically normal appearing white matter and LPA in the evaluation of cortical dyslamination. These methods may be translatable to the *in vivo* detection of mild cortical malformations.

## Introduction

A proportion of localized structural brain abnormalities and malformations causing epilepsy remain “occult” preoperatively, with current magnetic resonance imaging (MRI) technologies. These include cases of pathologically confirmed focal cortical dysplasia (FCD) type II 33 as well as milder malformations of cortical development [mild malformations of cortical development (MCD)] 3, 22. Advanced MRI techniques have the potential to improve the identification and characterization of these lesions. This improvement, however, is dependent on a thorough understanding of the cellular basis of these techniques, best achieved by correlating the lesion's magnetic resonance (MR) and its pathology directly with each other 22. High‐resolution MRI (7T and 9.4T) of tissue samples followed by histology has been utilized to study post‐mortem (PM) cortical alterations in multiple sclerosis 17, 27, Alzheimer's disease 14, 29, schizophrenia 18 as well variations in normal brain myelo‐architecture and cyto‐architecture 10, 16, 34 including epilepsy surgical samples 11, 12. Many of these previous studies have used traditional Nissl and myelin stains for pathological qualitative or quantitative analysis 10, 12, 16, 34. With the application of immunohistochemistry to specific cellular and structural components of cortical laminae and white matter, there is further potential to dissect out the cellular basis of MRI signal alterations in “cryptogenic” focal epilepsies. Furthermore, varying reactive pathological changes are frequently noted in surgical epilepsy specimens, including gliosis, inflammation and blood–brain barrier breakdown 1, 32, which could potentially also influence MRI signals and need to be accounted for.

Previous quantitative neuroimaging and pathology correlative studies have generally relied on the analysis of small regions of interest (ROIs) in tissue samples 9, 21, which is dependent on accuracy of co‐registration between sample and MRI 8 and may not be representative of the entire pathology, particularly in more ill‐defined lesions as mild MCD. Whole slide quantitative analysis methods (WSA) maximize the tissue areas analyzed, and we have recently shown this to be a more reliable quantitative application in the study of mild MCD 19. Confirmed pathology–imaging correlations based on *ex vivo* high‐field MRI of tissue specimens have the potential to be translated to the *in vivo* characteristics of these lesions and ultimately to improve detection, and potentially delineate the boundaries of these lesions, prior to surgery 22.

We present a series of cases where high‐field 9.4T MRI and quantitative analysis has been carried out on surgical samples from patients with focal epilepsy including histologically normal and dysplasia cases. We employed quantitative immunohistochemistry for a broad range of neuronal, glial, microglial, myelin and vascular markers, including the application of WSA analysis, in order to further explore the relationship between pathological features and MR signal change in epilepsy cortical resections. Our aims were to identify quantitative MRI (qMRI) measures that could be of predictive value in the preoperative evaluation of focal epilepsy, in particular FCD types and mild MCD.

## Methods

### Case selection

The specimens were selected prospectively from adult patients undergoing surgical treatment for epilepsy at the National Hospital for Neurology and Neurosurgery, Queen Square, during the period 2011–2013. Twelve cases were studied after obtaining preoperative consent for use of surgical tissue, and the study has approval through the ethics committee of the UCL, Epilepsy Society Brain and Tissue Bank. To correlate these samples with a reference, normal control tissue, a further sample from a PM of a patient without cortical neuropathology or history of epilepsy was included. The clinical details including epilepsy history and preoperative MRI diagnosis are presented in Table 1. The preoperative suspected diagnosis included FCD subtypes (five cases), hippocampal sclerosis 1 and dysembryoplastic neuroepithelial tumor 1. The resections were from the frontal lobe (eight cases), temporal lobe (three cases), parietal lobe (one case) and occipital lobe (one case); the control PM tissue was from the temporal lobe.

**Table 1:** Clinical data of patients and fixation timesAbbreviations: DNT = dysembryoplastic neuroepithelial tumor; EEG = electroencephalogram; FCD = focal cortical dysplasia; FS = febrile seizures; GS = generalized seizures; HS = hippocampal sclerosis; icEEG = intracranial EEG from depth electrodes; ILAE = International League Against Epilepsy; LPA = line profile analysis; MRI = magnetic resonance imaging; NA = not applicable; PM = post‐mortem; qMRI = quantitative MRI; SE = status epilepticus; Sz = comprises focal seizures with and without loss of consciousness

Case	Age onset epilepsy (years)	Age at surgery (years)	Duration of epilepsy (years)	Seizure types	Gender	Preop MRI diagnosis	Localization of resection	Postoperative outcome	Fixation time* (days)	Used in study
1	8	31	23	Focal Sz, GS	F	Extensive malformation with subcortical changes	Right frontal	Reduced frequency of seizures	452 (T2, T2*) 464 (T1)	qMRI
2	30 (FS—2 years)	54	24	Focal Sz	F	Left HS: left hemisphere smaller	Left temporal lobectomy	Not seizure free: new right HS	142 (T2, T2*) 152 (T1)	qMRI
3	15	32	17	Focal Sz, GS	M	FCD in calcarine fissue	Left temporal lobectomy (icEEG guided)	Not seizure free Psychiatric illness	122	qMRI
4	3	27	24	Focal S	F	Minor ventricular prominence	Left frontal resections (icEEG guided)	Seizure free	378	qMRI LPA
5	25	58	33	Focal Sz	F	FCD in left middle frontal gyrus, extending to ventricular surface	Left frontal resection (icEEG guided)	Seizure free	21	qMRI
6	14	49	34	Focal Sz	M	Previous surgery for DNT residual tumor	Left frontal lobe	Seizure free	10	qMRI LPA
7	11	23	12	Focal S, GS, SE	M	No FCD	Left frontal resection (icEEG guided)	Seizure free	5	qMRI
8	8	24	16	Focal Sz, GS	F	No specific abnormality	Left frontal resection (icEEG guided)	ILAE 1b (some minor seizures)	23	qMRI LPA
9	16	32	16	Focal S	M	Blurring of the right frontal lobe	Right frontal, basal resection (icEEG guided)	Seizure free	29 (sample 1) 36 (sample 2)	qMRI LPA
10	7	28	21	Focal Sz	M	FCD in right supra‐marginal gyrus	Right parietal (icEEG guided)	One seizure since operation	8 (sample 1) 10 (sample 2)	qMRI LPA
11	NA—no epilepsy	62 (PM control)	NA	NA	F	No cortical lesion, salivary gland tumor	NA	NA	568	qMRI LPA
12	18	30	12	Focal Sz, GS	M	Normal	Right frontal (icEEG guided)	ILAE 2b	122 (T2) 132 (T1)	qMRI
13	11	23	12	Focal Sz, GS		Perinatal infarct/FCD IIId	Right occipital lobe	Two seizures following operation—now seizure free for 8 months		LPA

Seizure outcome is taken at the time point for evaluation in this study.*The fixation time is recorded as time from resection to MRI scan acquisition.

### Sample preparation

In each of the 13 cases, a section of cortex and underlying white matter of 5‐mm thickness was sliced using a cradle as previously described 8 and fixed for a minimum period of 5 days in formalin. Either one or two such samples from each case were selected for 9.4T imaging, aiming to include macroscopically pathological and normal cortex as well as white matter in all cases. The sample was fixed rigidly in a tissue‐tek^®^ (Sakura, Tatcham, UK) cassette, which was held tightly within a plastic tube to minimize movement artifacts and filled with fomblin^®^ perfluoro polyether (LC 08, Solvay Solexis, Milan, Italy) to avoid susceptibility artifacts. In a previous pilot study carried out on PM tissues, there were no adverse effects of the fomblin medium on tissue processing and immunostaining protocols.

### MRI protocol

The samples were scanned on a 9.4T MR scanner (Agilent Technologies, Santa Clara, CA, USA) using a volume coil with a diameter of 33 mm (Rapid Biomedical GmbH, Rimpar, Germany). The applied overnight MRI protocol consisted of T2‐weighted images and T1, T2, T2* and magnetization transfer ratio (MTR) maps. Multi‐slice spin echo sequences were used for the acquisition of these data except for the T2* and MTR maps where multi‐slice gradient echo sequences were applied. After manual global shimming and acquiring fast localization images, 12 slices were acquired using a field of view of 35 × 35 mm^2^, an in‐plane spatial resolution of 136 × 136 μm^2^ and a slice thickness of 500 μm. Depending on the contrast, the echo time (TE) and the repetition time (TR) were:

(i)  T2‐weighted images: TE = 55 ms, TR = 4500 ms and the acquisition time (TA) was 96 minutes.
(ii)  T1 maps: TE = 10 ms, TR = 0.3/0.5/0.8/1.3/2/4 s and TA = 145 minutes.
(iii)  T2 maps: TR = 2 s, TE = 12.6/20/35/60 ms and TA = 102 minutes.
(iv)  T2* maps: TR = 290 ms, TE = 5.2/7/9/11/13/20 ms, flip angle (FA) = 40° and TA = 75 minutes.
(v)  MTR maps: TR = 260 ms, TE = 5 ms and FA = 40°. Two frequency offsets of the saturation RF pulse were used: 6 kHz (MT weighted) and 100 kHz (control). TA = 65 minutes.

The total scanning time for the entire protocol was approximately 8 h. T1, T2, T2* and MTR maps were obtained by processing the data using both an in‐house developed script running under Matlab (Mathworks, Massachusetts, USA) and ImageJ (National Institutes of Health, Bethesda, MD, USA; http://imagej.nih.gov/ij/index.html).

### Immunohistochemistry and quantitation on ROIs

The tissue samples were then routinely processed, sections cut at 7 μm and an immunohistochemistry panel applied as detailed in Table 2. All sections were assessed qualitatively for the pathology present on the scanned section as detailed in Table 3. Between three and four ROIs were outlined on each of the pathology sections to include normal, as well as pathological, white matter and cortex as detailed in Table 3. The histological sections were manually spatially aligned with the MRI‐scanned images, matching anatomical landmarks in the section, corresponding ROIs identified and qMRI values for T1, T2, T2* and MTR calculated for each area. Quantitative evaluation of immunohistochemistry staining was carried out on neurofilament (SMI32), myelin basic protein (MBP/SMI94), microtubule‐associated protein 2 (MAP2), synaptophysin, microglial marker CD68, albumin (for loss of integrity of blood–brain barrier) and GFAP (for astrogliosis) stained sections with Image Pro Plus software (Media Cybernetics, Buckinghamshire, UK). Using RGB thresholding, the immunostaining reaction was detected in all fields within the ROI, captured sequentially at ×40 objective (Nikon Eclipse microscope and camera). An average of 71 high power fields (SD 25) were captured for white matter ROI and 69 fields (SD 27) for gray matter. A mean labeling index (LI) was then calculated for each ROI. Repeat measurements were carried out on MAP2, SMI94 and GFAP section (between 12 and 30 ROI), which showed reproducibility; intraclass correlation coefficients of 0.81, 0.67 and 0.88, respectively. NeuN was not included in quantitative analysis due to the noted variability in the intensity of staining with longer fixation times 20; CD34‐ and nestin‐labeled sections were also not evaluated quantitatively due to the overall paucity of positively labeled cells.

**Table 2:** Immunohistochemistry panelAbbreviations: BC = balloon cells; DN = dysmorphic neurons; FCD = focal cortical dysplasia; GFAP = glial fibrillary acidic protein; LEAT = long‐term epilepsy associated tumor; LPA = line profile analysis; MAP2 = microtubule‐associated protein 2; ROI = region of interest study where small areas of interest were analyzed; WSA = whole slide analysis study

Antibody	Structure/cells labeled	Source; (dilution) method	Utilization in current study*
Myelin basic protein (SMI94)	Myelinated axons	Sternberger monoclonals; 1/2000	ROI, WSA, LPA
NeuN	Neuronal marker	Chemicon; 1/2000	LPA
Neurofilament: non‐phosphorylated (SMI32)	Pyramidal cell bodies (layer III, V; normal cortex), axons; in FCD type II, DN	Sternberger monoclonals; 1/500	ROI, LPA
Neurofilament: phosphorylated (SMI31)	Axons; in FCD type II, DN	Sternberger monoclonals (USA); 1/5000	LPA
Synaptophysin	Synaptic density	Dako; 1/100	ROI, LPA
MAP2	Neuronal cell body and dendrites	Sigma; 1/5000	ROI, WSA, LPA
Albumin	Regions of blood–brain barrier breakdown	Dako; 1/80 000	ROI
GFAP	Reactive cellular and chronic fibrillary gliosis	Dako; 1/2500	ROI, LPA
Nestin	Acute gliosis/BC	Chemicon; 1/8000	LPA
Calbindin	Interneurons (cortical layers II–III mainly)	Abcam; 1/40	LPA
CD68	Microglia	Dako; 1/100	ROI, qualitative
CD34	Vasculature: tumor cells in LEAT, BC in FCD	Leica/Novocastra; 1/50	Qualitative

*Indicates which markers were used in different studies.

**Table 3:** Neuropathological diagnosis represented in resections, the whole sections subjected to 9.4T MRI and regions of interest (ROI) for analysisAbbreviations: BC = balloon cells; DN = dysmorphic neurons; FCD = focal cortical dysplasia; HS = hippocampal sclerosis; ICE injury = intracranial electrode injury site; ILAE = International League against Epilepsy; MRI = magnetic resonance imaging; PAS = periodic acid schiff stain; WM = white matter

Case	Sample number	Underlying pathology diagnosis	Pathology represented				
			Whole section (9.4T MRI)	ROI 1	ROI 2	ROI 3	ROI 4
1	1	Extensive FCD type IIB “skip lesions” with lamination preserved in regions. Few BC and myelination qualitatively normal ICE injury (22 days*)	FCD IIB ICE injury	WM–FCD II	WM–normal	Cortex–FCD II	Cortex–ICE injury
2	1	HS (ILAE type 1): no cortical lesion	Path negative	WM–normal	WM–normal	Cortex–normal	—
3	1	No lesion	Path negative	WM–normal	WM–normal	Cortex–normal	—
4	1	FCD IIA with PAS‐positive dysmorphic neurons	FCD IIA	WM–normal	Cortex–normal	WM–FCD	Cortex–FCD
5	1	FCD IIB	FCD IIB	WM–normal	Cortex–normal	WM–FCD (Few BC)	Cortex–FCD (few DN)
6	1	Dysembryoplastic neuroepithelial tumor ICE injury (3 days*)	No tumor ICE injury	WM–normal	Cortex–normal	WM–ICE injury and increased normal neurons	Cortex–ICE injury
7	1	FCD IIA with PAS‐positive dysmorphic neurons	Path negative	WM–normal	WM–normal	Cortex–normal	—
8	1	No lesion	Path negative	WM–normal	WM–normal	Cortex–normal	—
9	1	Focal FCD IIA	Path negative	WM–normal	WM–normal	Cortex–normal	
	2		Increased WM neurons	WM–abnormal but no DN	Cortex–normal		—
10	1	FCD IIB ICE injury (14 days*)	ICE injury	WM–normal	WM–normal	Cortex–ICE injury	—
	2		FCD IIB	WM–FCD	Cortex–FCD IIB (some DN but not full thickness)	—	—
11		Post‐mortem control case. No FCD/pathology	Path negative	WM–normal	WM–normal	Cortex–normal	
12		Focal FCD IIA	Path negative	WM–normal	WM–normal	Cortex–normal	

*The age of the electrode injury site is recorded as the time between electrode implantation and resection of tissue.

### Whole slide scanning

Based on initial findings from the ROI analysis, SMI94/MBP‐ and MAP2‐stained sections from each case were selected for whole slide scanning analysis. Slides were scanned with a Leica SCN400F digital slide scanner (Leica Microsystems, Wetzlar, Germany); images were processed into a Pyramid TIFF file, stored on a fileserver and viewed and managed on the SlidePath Digital Image Hub (Leica). Analysis was carried out with Definiens Tissue Studio 3.6 (Definiens AG, Munich, Germany) as follows. The entire area of white matter was outlined on each image using a stylus pen on a touch‐sensitive computer screen, drawn at a set distance of 0.5 mm from the cortical margin, to exclude layer VI neurons. The mean area was 29.4 mm^2^ (range 7–63 mm^2^) for MAP2 and 40 mm^2^ (range 13.5–84 mm^2^) for SMI94/MBP. The intensity threshold for detection of immunostaining was preset in a selected area then the entire image was processed and the LI calculated for the entire outlined white matter.

### Line profile analysis (LPA) in the assessment of lamination

In seven cases 4, 6, 8, 9, 10, 11, 13, LPA was carried out. Regions of normal laminated cortex were selected and/or a region (cases 4, 10 and 13) with dysplasia and dyslamination. On all immunostained sections, images of the full cortical thickness were captured at ×4.7 magnification, converted to gray scale and the intensity scale of pixels along a vectors perpendicular to the pial surface through all cortical layers were analyzed with Image Pro Plus software. In addition, the approximate distance of each cortical layer from the pial surface was measured on NeuN‐stained sections of normal laminar cortex. Similar LPA of matched cortical regions was carried out on all MRI sequences.

Statistical analysis was carried out with SPSS (version 21 for Windows, IBM Corporation, Armonk, NY, USA) using nonparametric correlation tests for the comparison of MRI and pathology measures (Spearman's correlation and Mann–Whitney test).

## Results

The pathological diagnosis on the sections selected for 9.4T was FCD IIa in one case, FCD IIb in two cases, FCD IIId in one case and “pathology negative” (no focal lesional pathology) in nine cases; in three cases intracranial electrode (ICE) injury sites were also present (Table 3).

### Quantitative pathology and MRI measurements

**Figure 1:**
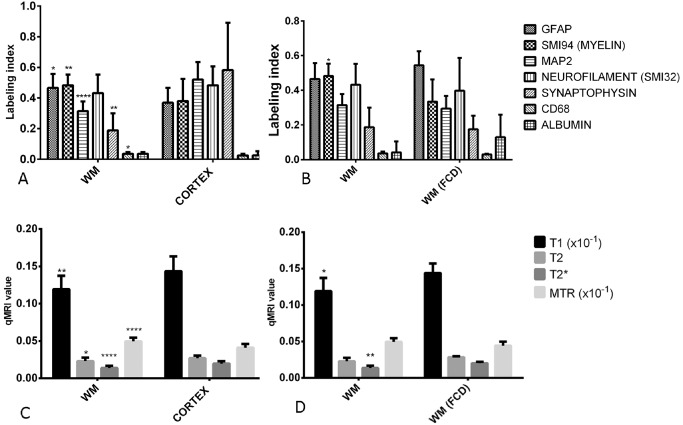
*Bar graphs of comparison of quantitative pathology values in different regions of interest and quantitative magnetic resonance imaging (* *MRI* *) values*. **(A)** Pathology measures between normal cortex and white matter and **(B)** normal white matter and white matter in the region of focal cortical dysplasia (FCD). **(C)** MRI measures between normal cortex and white matter and **(D)** normal white matter and FCD white matter. Mann–Whitney test used for comparison between groups and the significant values shown as *P < 0.05, **P < 001, ****P < 0.0001. MRI units for T1, T2 and T2* in seconds; magnetization transfer ratio (MTR) is a ratio value.

There were 43 ROI analyzed in all; 24 in white matter and 19 in cortex with 31 in histologically normal appearing areas and 12 in pathological areas. There were significant differences in quantitative pathology measures between normal cortical ROI and normal white matter ROI for GFAP (*P* < 0.05), SMI94/MBP (*P* < 0.001), MAP2 (*P* < 0.0001), CD68 (*P* < 0.05) and synaptophysin (*P* < 0.001) but not for neurofilament or albumin (Figure 1A). When these measures were compared between normal white matter ROI and FCD white matter ROI, a significant reduction was observed for SMI94/MBP (*P* < 0.05) (Figure 1B); although the number of FCD cases is small, this finding is in keeping with previous observations of reduced myelin in dysplastic region 28. There were no differences for normal cortical ROI and FCD cortical ROI. For qMRI measures, significant differences were observed for T1 (*P* < 0.01), T2 (*P* < 0.05), T2* (*P* < 0.0001) and MTR (*P* < 0.0001) between cortex and white matter ROI (Figure 1C), between normal white matter and FCD white matter for T1 (*P* < 0.05) and T2* (*P* < 0.01) (Figure 1D) and between normal cortex and FCD cortex for MTR (*P* < 0.05).

### Quantitative pathology and MRI correlations

#### Myelin

**Figure 2:**
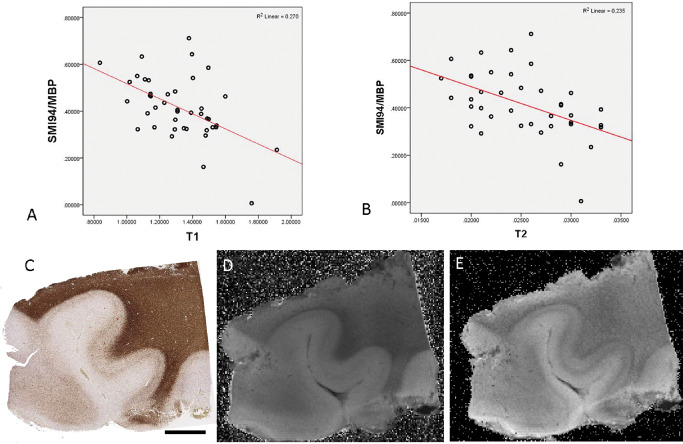
*Quantitative pathology and magnetic resonance imaging (* *MRI* *) correlations with SMI94/myelin basic protein (* *MBP* *) marker*. Significant inverse correlations were observed with **(A)** T1 and **(B)** T2 values overall MRI **(C)** case 9 (pathology negative) section stained with SMI94/MBP stain and **(D)** T1 and **(E)** T2 sequences on 9.4T MRI. Bar = 5 mm.

In SMI94/MBP‐stained sections there was a significant inverse correlation between the LI and qMRI measures in the 43 ROI for T1 (*P* < 0.005) (Figure 2A,C,D), T2 (*P* < 0.005; Spearman's correlation) (Figure 2B,E) and T2* (*P* < 0.05); there was a positive correlation between SMI94/MBP and MTR (*P* < 0.05). When considering normal white matter ROI alone, a significant inverse correlation between SMI94/MBP and both T1 and T2 was still observed (*P* < 0.05). With WSA, there was also a significant inverse correlation between SMI94/MBP and T2 (*P* < 0.05).

#### Neuronal density

**Figure 3:**
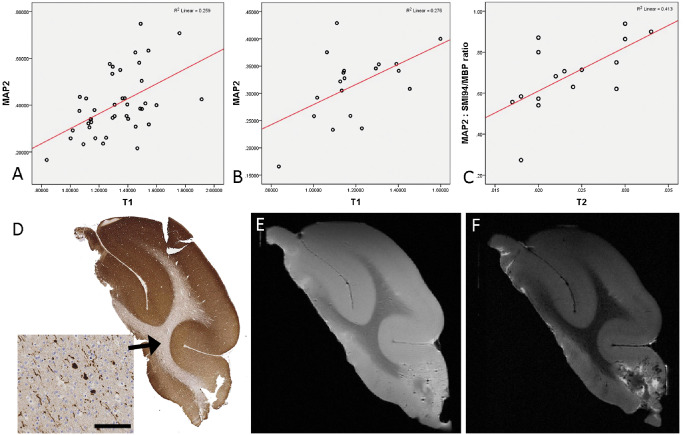
*Quantitative pathology and magnetic resonance imaging (* *MRI* *) correlations with microtubule‐associated protein 2 (* *MAP* *2)*. There was a significant positive correlation with T1 in all 43 regions of interest (ROI) (*P* < 0.005, Spearman's correlation) **(A)**. There was a significant positive correlation with T1 in normal white matter regions of interest (*P* < 0.05, Spearman's correlation) **(B)**. When ratios of microtubule‐associated protein 2 : SMI94/MBP were calculated for, there was a significant positive correlation overall ROI for all MRI sequences (T1, T2, T2* and MTR; *P* < 0.01 to *P* < 0.0001 see text) and as shown here for normal white matter ROI alone with T2 (*P* < 0.01) **(C)**. **(D)** MAP2 stained section of case 2 (pathology negative; inset shows the single white matter neurons and processes within the normal white matter. **(E)** Case 2 with T1‐ and **(F)** T2‐weighted MRI sequences at 9.4T. Bar = 5 mm in **D** (∼300 μm).

In MAP2‐stained sections, there was a significant positive correlation between the LI and qMRI measures over all 43 ROI for T1 (*P* < 0.005) (Figure 3A,D,E) and T2* (*P* < 0.05). When considering normal white matter ROI alone, there was also a positive correlation between MAP2 LI and T1 (*P* < 0.05) (Figure 3B). With WSA for pathology‐negative cases, there was a positive correlation between MAP2 LI and T2*, T2 (*P* < 0.05) (Figure 3F) and an inverse correlation with MTR (*P* < 0.01). There were no significant correlations noted for neurofilament LI and MRI measures.

When the MAP2 and SMI94/MBP LI were expressed as a ratio, over the 43 ROI there was a more significant positive correlation with T1 (*P* < 0.0001), T2 (*P* < 0.001), T2* (*P* < 0.01) and negative correlation with MTR (*P* < 0.01); with normal white matter ROI alone there was a positive correlation between the MAP2/SMI94 ratio with both T1 and T2 values (*P* < 0.01) (Figure 3C).

#### Reactive pathological changes

There was a positive correlation between T2 measures in the normal cortical ROIs and GFAP LI (*P* < 0.05). There was also a positive correlation between albumin labeling in normal appearing cortex and T1 measures (*P* < 0.05). Albumin was qualitatively observed to stain the neuropil adjacent to vessels and some reactive astrocytes, as previously described 31. Correlations were not seen for GFAP or albumin in other ROI classes, and no significant correlations were noted for microglial marker CD68.

**Figure 4:**
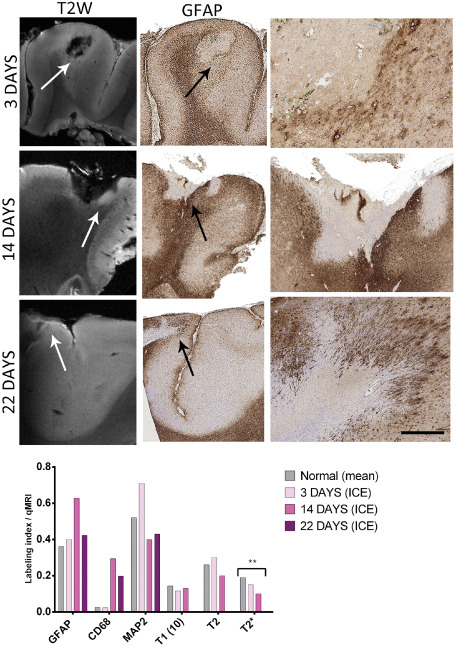
Intracranial electrode (ICE) injuries. In three cases ICE injury sites were represented within the tissue blocks scanned at 9.4T magnetic resonance imaging. The ages of these ICE injuries (time from electrode insertion to the time of tissue resection) were 3, 14 and 22 days, representing acute to subacute stages of cellular organization. The position of the injury is arrowed in T2 image with evidence of intensity at the margin of the injury cavity noted. The ICE injury site is shown on the GFAP stain (with the cavity indicated by a black arrow), indicating the reactive cellular margin of astrocytes (shown in high magnification in the corresponding column on the right side). Graphical representation of values for the region of interest corresponding to the ICE injury is shown in the bar chart with an increase in GFAP and CD68 labeling index (LI), decline in microtubule‐associated protein 2 LI and T2* alterations with age of the ICE injuries. (T2 and T2* values are shown at ×10^1^). Bar equivalent to ∼1000 μm in middle column GFAP and ∼50 μm in right hand column GFAP).

In three cases, localized ICE injuries were identified in the sections submitted for 9.4T; the age of the injuries, taken as the time from electrode insertion to tissue resection, were 3 days (case 6), 14 days (case 10) and 22 days (case 1). The high frequency of ICE injuries in this cohort is accounted for by the high proportion of cases that had undergone intracranial monitoring for “MRI‐negative” epilepsy. The injury site was clearly visible as a small empty hypo‐intense “cavity” in the cortex on MRI, and in the marginal zones on T2, there was impression of increased signal from 3‐ to 14‐day‐old injuries (Figure 4). Quantitative evaluation of CD68 showed a trend for increased CD68 (*P* = 0.064 Mann–Whitney) in these lesions compared with normal cortex, a decline in MAP2 (*P* = 0.07, Mann–Whitney) but no significant alteration in GFAP with days post‐injury in this small group (Figure 4). There was a significant reduction in T2* values in injured compared with normal cortex (*P* = 0.02) (Figure 4).

#### Clinical correlations

There were no significant correlations between MRI and pathology quantitative parameters in relation to age of epilepsy onset, duration of epilepsy or localization of the resection. There was also no effect of fixation times on MR measurements; there was an inverse correlation between fixation time and MAP2 and SMI32 staining (*P* < 0.032 Spearman's) but not for the other markers, including SMI94/MBP.

### LPA

#### Normal laminar cortex

**Figure 5:**
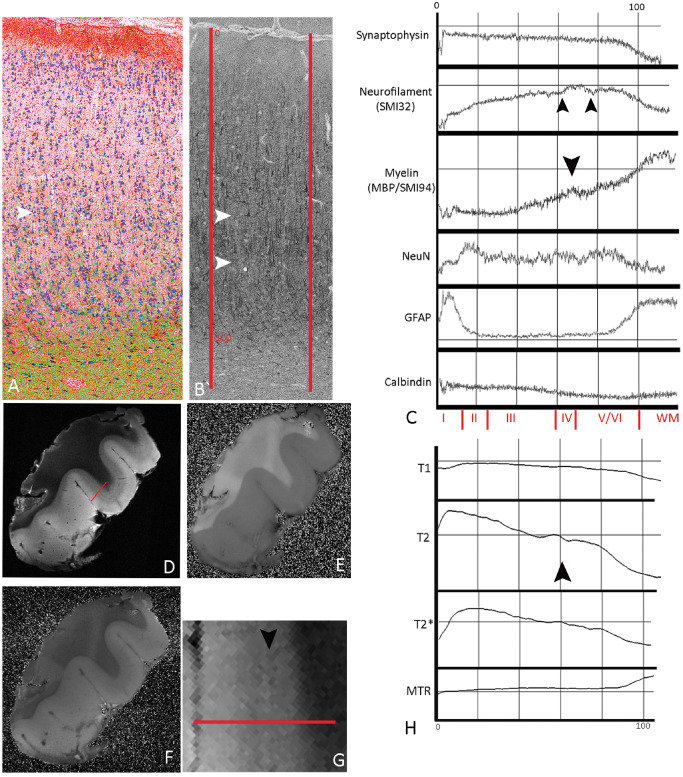
*Line profile analysis (* *LPA* *) in normal cortex (case 8)*. **(A)** Merged and superimposed images, recolored for illustrative purposes, of NeuN (blue), myelin (green) and GFAP (red); same area is shown in **(B)** labeled with neurofilament/SMI32 in gray scale. Gray scale monochrome images were used for LPA along vectors drawn at an axis perpendicular to the pial surface through all cortical layers (indicated with the red lines SMI32/neurofilament stain in **B**). **(C)** Graphical representation of LPA of mean intensities of labeling between the two vectors in this case; the *x*‐axis represents the distance through the cortex scaled as 0 = the pial boundary to 100 = cortical white matter boundary (shown in red on panel **B** and the approximate position of cortical layers, as measured with image pro plus on NeuN‐stained sections, is shown at the bottom of graph in **C** in red text). Intensity of gray‐scale signal is shown in the *y*‐axis (note: the graphs have been inverted for illustration so higher *y*‐value corresponds to lower gray scale/greater immuno‐positive reaction). *Synaptophysin* shows a relatively flat intensity line profile through the cortex until the white matter junction; *neurofilament (SMI32)* shows greater immunostaining intensity in deeper compared with superficial cortex with irregularities in the line profile in the mid‐layers (double arrows in **B** and **C**) reflecting the zones between the pyramidal cell dendrites in layers III and IV and V. The profile for *MBP* */SMI94* also shows increased intensity of immunostaining on LPA in deeper cortical layers corresponding to the radial fibers, with an impression of a small peak in mid‐layers (arrow in **A** and **C**), which is likely detecting the horizontal myelinated fibers. NeuN LPA is low in cell‐free layer I and shows peak intensity in LPA in layer II, corresponding to the greater neuronal packing density of small neurons, whereas the neurons in deeper cortical layers are more widely spaced and the line profile flattens off until the white matter. GFAP LPA intensities peaked in layer I (corresponding to the Chaslin's gliosis) and in the white matter. Calbindin showed lower overall intensities, due to the overall low density of immunopositive cells, but with highest intensities in outer cortical layers (I–III). MRI sequences are T2 [**D**; bar indicates region of LPA and shown in higher magnification in **(G)**], MTR **(E)** and T1 **(F)**. **(H)** LPA of MR sequences in T2 and T2* and to some extent T1 show higher intensities of LPA in superficial cortical layers with a small peak in mid‐layers on T2 (arrow) approximately corresponding to the region of altered intensity in the neurofilament and myelin sections in the region of layer IV.

LPA of normal cortex and white matter with neuronal‐ and layer‐specific markers are illustrated in case 8 (Figure 5); NeuN showed a peak in layer II corresponding to the high packing density of small neurons (Figure 5A,C) whereas neurofilament confirmed increased labeling in lower compared with superficial cortical layers (Figure 5B,C) with a small fluctuation in mid‐cortical layers, reflecting the region between the tramline‐like bands of labeling of the dendrites of the pyramidal neurons in layers III, V and VI with their high neurofilament content. Synaptophysin by contrast showed a flat labeling intensity profile through the cortex reflecting the more homogenous staining with a relatively sharp drop at the cortical–white matter boundary (Figure 5C). LPA for SMI94/MBP showed increased intensity in deeper cortical layers corresponding to the radial fibers with a small wave in mid‐layers (layer IV) likely reflecting horizontal myelinated fibers (Figure 5C). GFAP line profiles were highest in layer I and white matter corresponding to the Chaslin's gliosis and white matter gliosis, respectively (Figure 5A,C). Calbindin showed lower intensities overall, reflecting low density of labeled neurons, but higher in outer cortical layers (I–III) compared with deeper cortex in keeping with cell distribution (Figure 5C). LPA of normal cortex in cases 4, 6, 9 and 11 (Figures S1–S4b) were broadly similar: alterations in line profile intensity in myelin stains (MBP/SMI94) in mid‐layers correlating to horizontal myelinated fibers were more apparent in some cases (case 9; Figure S2 and case 11; Figure S3) than others (case 6; Figure S1). Similarly with neurofilament stains, waves in the LPA intensity in mid‐layers, correlating to the zone between dendrites of pyramidal layers III and V, were prominent in cases 6, 9 and 4 (Figures S1, S2 and S4b) but less pronounced in others (case 11; Figure S3). These differences between normal cases likely reflect normal regional, anatomical variations in cyto‐ and myelo‐architecture.

LPA in MRI sequences confirmed the qualitative impression of increased signal intensity in outer cortical layers in T2 as previously described 12 (Figure 5D,G) and to a lesser extent in T1 (Figure 5F,H). In addition, waves in the LPA intensity were observed in mid‐cortex (Figure 5H) and likely correspond to the profiles observed in neurofilament and myelin‐stained sections in layer IV region. The MTR showed a more uniform, flat line profile through the cortex, changing at the white matter. LPA in further cases of normal cortex (Figures S1–S4b) supported these findings, with corresponding fluctuation in the profile intensity of T2 and T2* sequences in mid‐cortex.

#### Dyslaminar cortex in FCD

**Figure 6:**
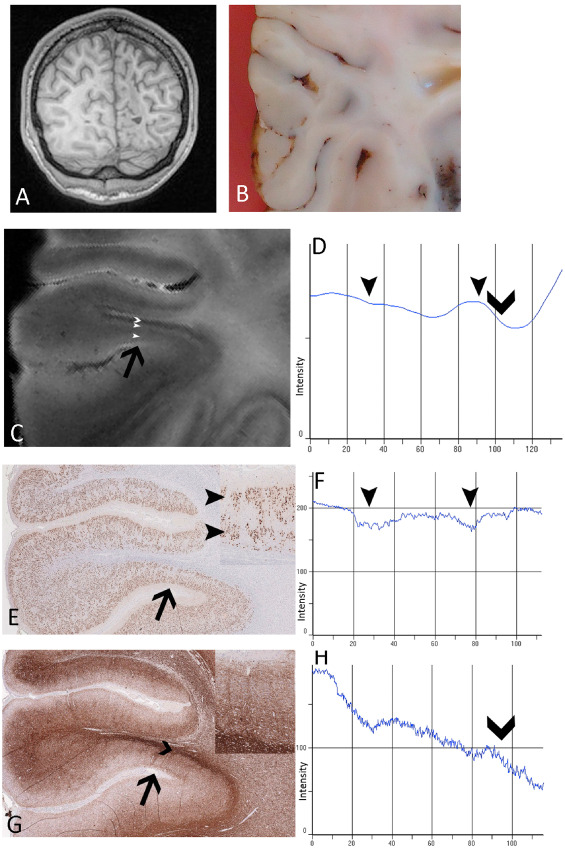
*Line profile analysis (* *LPA* *) in case 13 with focal cortical dysplasia‐type iiid*. **(A)** *In vivo* magnetic resonance imaging (MRI) showing right occipital “ulegyric” malformation and **(B)** post‐fixed resected surgical specimen prior to 9.4T MRI. **(C)** 9.4T MRI T2 image illustrating one malformed gyrus and corresponding gyrus shown on sections stained with **(E)** NeuN **(G)** and myelin basic protein/SMI94 **(G)**; black arrow in each indicates the region for LPA (shown at higher magnification in insets in **E** and **G**). The outermost layers are hyperintense on T2 and two peaks of hyperintensity (shown as arrowheads on **C**, **D**) correspond to a superficial and deep neuronal layer (shown with arrow heads on **E** in inset, and on **F** as dips in LPA intensity corresponding to the greater immunolabeling). A deeper hypointense band (marked with chevron on **C** and **D**) corresponds to increased myelinated fibers in the deep cortex/subcortical region (shown with chevrons on **G** and on **H** as a fall in intensity due to increased labeling). In the graphical representation of LPA of mean intensities (**D**, **F** and **H**), the *x*‐axis represents the distance through the cortex, scaled as 0 = the pial boundary to 100 = cortical white matter boundary. **E** and **G** are shown at ×4 magnification.

LPA of dysplastic cortex with dyslamination is illustrated by case 4, 10 and 13 (Figure 6). In case 4, preoperative *in vivo* MRI was normal, resected frontal lobe was macroscopically unremarkable (Figure S4A) but FCD IIA confirmed histologically in the depth of one sulcus, with numerous dysmorphic neurons scattered within the cortex but absent balloon cells (Figure S4A). LPA carried out on the FCD region compared with adjacent normally laminated cortex showed lower overall intensity values for NeuN (Figure S4A) and altered line profile for MAP2 (Figure S4B,C) in FCD, correlating with reduced neuronal labeling and reflecting abnormal neuronal density and distribution, in keeping with previous stereological quantitative studies in FCD type II 30. Similarly, LPA of T2 and T2* (Figure S4A,B,C) also showed differences in intensities between the normal and FCD regions, but there were no obvious differences for T1 and MTR (Figure S4B,C). These observations on LPA were also reflected in the qMRI values for this region of FCD IIA that showed lower T2 and T2* values compared with both the adjacent cortex as well as mean values for normal cortex in the whole series. In case 13 with a perinatal infarct and FCD type IIId (Figure 6A,B), with severe abnormalities of the cortical myeloarchitecture and cyto‐architecture (Figure 6E,G), LPA of T2‐weighted images (Figure 6C) showed distinct lamination corresponding to the abnormal neuronal and myelin cortical layering. LPA in a third case with dysplasia (FCD IIB) (case 10; Figure S5) also showed abnormal line profiles, with flatter contours observed on NeuN‐, myelin‐ and neurofilament‐stained sections as well as T2*, T2 and T1, reflecting abnormal cortical lamination and myelo‐architecture.

## Discussion

Further technical advances in MRI, including increased field strength, improvements in image resolution and post‐processing quantitative analysis, are likely to improve future detection of subtle FCD and mild malformations in epilepsy 22. Such advances, however, need to be validated with comparative neuropathology, still regarded as the gold standard in the diagnosis and classification of lesion‐based epilepsies 3, 22. In this study we have aimed to address this need through correlating *ex vivo* 9.4T qMRI measures with quantitative immunohistochemistry. We have shown significant correlations between MRI measures and myelin and neuronal density measures, even in histologically “normal” appearing white matter. Such correlations show the potential of high‐resolution MRI in the detection of FCD and mild MCD with subtle white matter pathological changes.

### FCD

Established *in vivo* MRI hallmarks of FCD type II include thickening of the cortex, a less distinct gray–white matter interface and increased signal intensity in T2/FLAIR, with reduced or hypointense T1 signals in the white matter 5, 22, 33. It is recognized that not all confirmed FCD II show these alterations and there is evidence for improved MRI detection of FCD with the application of quantitative methods 2, 33. The pathological correlate of white matter T2 and FLAIR signal abnormalities is considered to reflect reduced myelin content 3, 6. Hypomyelination of the white matter in the region of FCD II is a common, but not essential, histological feature 23, confirmed through quantitative immunohistochemistry in more subtle cases 23, 28. The etiology of white matter hypomyelination is less well understood; it may represent a developmental deficit in local oligodendroglial maturation and myelination or reduced density of normally myelinated axons in the region of FCD 23, 28. In this current study in non‐FCD epilepsy cases, we demonstrated an inverse correlation between myelin immunolabeling and T2 over all ROI (including cortex and white matter) as well as a significant correlation when including only ROI from histologically unremarkable appearing white matter. This demonstrates the potential of 9.4T MRI to detect subtle differences in white matter myelination that could be implemented in the localization of occult FCD in patients with cryptogenic focal epilepsy as well as in the detection of other white matter myelination abnormalities recognized to occur in temporal lobe epilepsy 4, 11, 26. The present series also included a 3T “MRI occult” FCD II case, in which we confirmed a reduction in white matter myelin labeling and significant increase in T1 and T2* with 9.4T MRI in the dysplasia region with quantitative analysis compared with normal values.

### Mild MCD

Mild MCD encompass subtle (presumed developmental) cortical and white matter abnormalities and remain one of the more controversial entities described in focal epilepsies, both in view of their preoperative detection, histological criteria, biology and significance 3. Nevertheless they may represent a “biomarker” for focal epilepsy. In this study we confirmed a significant correlation between MAP2 neuronal labeling in the white matter and quantitative T1 measures, supporting the ability of 9.4T MRI to detect pathologically elevated white matter neuronal numbers. Mild MCD type II is currently poorly defined as an “excess” of single neurons in the white matter 3. In a recent quantitative analysis of 142 temporal lobe epilepsy cases utilizing WSS methods 19 we confirmed higher white matter neuronal densities in epilepsy patients (>2298 cells/mm^3^) compared with controls; however, the clinical significance of this finding, in terms of epilepsy control following surgery, remained unclear. In the current study we also implemented WSS, which we have shown a more efficient and reliable method for the quantitative analysis of larger ROI such as the white matter 19. We observed a correlation between white matter MAP2 neuronal labeling, T2*, T2 and MTR. This supports continued implementation and development of WSS technology for the rapid analysis of epilepsy lobectomy specimens. Indeed, limitations of the size of white matter ROI, which were necessary in our previous studies of *in vivo* 3T MRI/pathology correlations implementing stereology, may be one explanation for the lack of correlation between white matter neuronal number and qMRI then observed 9. Furthermore, in this current study we included MAP2 for the assessment of neuronal density as an alternative to previously employed NeuN marker. Threshold‐based analysis of the area of MAP2 immunostaining, which labels both neuronal cell body and dendrites, as an alternative to more traditional neuronal density measurements based on nuclear NeuN labeling 9, 19, may prove a more sensitive marker in the evaluation of altered white matter neuronal populations. Furthermore, Mild MCD II‐like pathologies are also recognized in other neurological conditions as schizophrenia, autism spectrum disorders 24, 35 as well as epilepsy and although its relevance, in terms of a prognostic marker, remains uncertain its *in vivo* detection may be important as a biomarker of abnormal neurodevelopment in these conditions.

### Previous qMRI studies

Our findings are in keeping with published *ex vivo* studies correlating qMRI and quantitative neuropathology measurements in other neurological conditions. Schmierer *et al* 27, in a study of PM tissues from patients with multiple sclerosis using 9.4T MRI, showed an inverse correlation between MBP and T2 in the cortex and an inverse correlation between MBP and T1 in the white matter. Spencer *et al* 29, in a mouse model of AD, showed that T1 correlated better with myelin density than cell density in the cortex. In a study of PM AD brains, Gouw *et al* also noted that increased white matter myelin loss was associated with increased T2 signal 14. In previous studies of 7T MRI of temporal lobe epilepsy samples, areas of in‐homogenous signal intensity in the white matter on T2 corresponded to fewer myelinated and unmyelinated axons on ultrastructural analysis 11. It is also plausible that the observed white matter microstructural alterations of reduced and abnormal white matter myelination 11, 23, 28 and increased neuronal density operate synergistically to influence MRI signal alterations. Indeed, in the current study, when we expressed MAP2 and myelin as a ratio, there was an augmented statistical association with qMRI measures.

### LPA and dyslamination

We utilized LPA to compare intensity gradients in normal laminar cortex and regions with histologically proven FCD types. Dyslamination (abnormal neuronal layering) is a hallmark of FCD type II and the only pathological abnormality in FCD type I 3. It can be evident on cresyl violet (Nissl) stained sections, although can be subtle with improved detection shown through application of cortical layer‐specific markers 15, 25, some of which were applied in the current study. Although less recognized, the cortical myelo‐architecture is also frequently altered in FCD, supported by previous quantitative studies 28 and is more dramatic in FCD type IIId as exemplified by the case in this series. Clarification of dyslamination in *in vivo* MRI could facilitate detection of subtle FCD and be utilized as a potential diagnostic technique for currently “occult” patients with electro‐clinically focal seizures. Previous MRI studies of human brain tissue samples have employed similar LPA methods. Fatterpekar *et al*. 10 imaged PM samples of the calcarine cortex at 9.4T and correlated this with Nissl and Luxol fast blue stains for myelin; they visualized bands of low signal in mid‐layers on MRI images corresponding to the myelinated fibers in the external band of Baillarger. Walters *et al* also confirmed the ability of high‐resolution MRI to detect cortical lamination in PM samples in regions outside the calcarine cortex 34 and noted that this correlated overall better with cortical myelo‐architecture than cyto‐architecture, as also in a study by Eickhoff *et al* 7. We were also able to visualize cortical lamination with MRI in a PM specimen taken from the temporal cortex, where the intracortical horizontal myelin plexus is less pronounced than in the visual striate cortex. Using similar LPA method, Garbelli *et al* 12 carried out an *ex vivo* 7T MRI of surgical epilepsy resections of FCD type III type a (temporal cortical dysplasia adjacent to hippocampal sclerosis). In normal cortex, they noted intensity differences on T2 between the upper and lower cortex separated by a dip of lower intensity signal correlating with cortical myelination patterns and the neuronal density of layer IV on NeuN sections; in FCD IIIa cases, however, this dip in mid‐layer intensity was lost and the T2 line profile through the cortex was flatter without a clear difference between supragranular and infragranular cortex 12. They concluded that altered LPA in dysplasia is strongly correlated with abnormal cortical myelination patterns.

With LPA we confirmed similar intensity profiles for T1 and T2 in normal cortex from varied cortical locations, with NeuN, neurofilament and MBP markers more consistently revealing contours and linear gradients across cortical layers and correlating with MRI line profiles. Furthermore, we observed differences in LPA on 9.4T MRI in histologically confirmed FCD II and FCD IIId reflecting the abnormal lamination, altered neuronal populations and abnormal myelo‐architecture. These studies support the potential application of LPA techniques in the identification and discrimination of FCD types.

### Reactive and secondary cortical changes

Gliosis is a ubiquitous finding in surgical resections from patients with epilepsy but of unknown cause. Furthermore, inflammatory changes and blood–brain barrier breakdown are reported that could influence MRI appearances 1. In our quantitative analysis of normal cortex, we noted a positive correlation between T2 and gliosis (GFAP) and albumin leakage and T1, suggesting that 9.4T MRI signals may be influenced by subtle reactive cellular changes.

There is a great incentive to understand processes of gliosis and brain repair responses, both the pathological mechanisms and MRI correlates, in particular the time course of early cellular changes, reparative neuro‐glial interactions as well as identifying any maladaptive, detrimental effects. We have previously used ICE “injuries” in patients with epilepsy as a model to study the time course of cellular responses and gliogenesis 13. In the current series we had three injuries of varying ages (acute to subacute) and although a small number, quantitative histological analysis confirmed the expected microglial responses accompanied by progressive loss of neurons and dendrites as highlighted with a reduction in MAP2 and a marginal astroglial reaction. Despite the gliotic margin and the qualitative impression of a rim of increased signal intensity around the injury cavity we noted a significant fall in T2* with days post‐injury compared with normal cortex. Although we have only three samples, it is likely that the T2* measure in these cases reflects the overall loss of neurons rather than the cellular glial reaction. Nevertheless, these cases highlight the potential of 9.4T MRI in the evaluation of this “model” of cortical injury; by including more ROI, representing zones of injury from the necrotic center to the penumbra as well as greater number and age of injuries, the sequence of cellular alterations in brain repair and imaging alterations could be teased out.

In conclusion, this study demonstrates the feasibility of quantitative 9.4T MRI to detect subtle differences in cortical and white matter neuronal numbers and myelination in histologically normal appearing cortex and white matter and LPA as a useful tool in the detection of subtle cortical dyslamination. These methods have the potential to be translated to the *in vivo* detection of mild MCD and occult FCD cases in epilepsy.

## Conflict of Interest

All authors have no conflicts of interest to declare.

## Supporting information


**Figure S1.** Line profile analysis (LPA) for case 6 (no cortical dyslamination).
**Figure S2.** Line profile analysis (LPA) for case 9 (no cortical dyslamination).
**Figure S3.** Line profile analysis (LPA) for case 11 (post‐mortem sample with no dyslamination).
**Figure S4.** (A) Line profile analysis (LPA) in FCD IIA (case 4).
**Figure S5.** Line profile analysis (LPA) for case 10 in FCD IIB.
