# Supplementary figures and images for: Combined Ex Vivo 9.4T MRI and Quantitative Histopathological Study in Normal and Pathological Neocortical Resections in Focal Epilepsy
Source: Brain Pathol. 2015 Sep 6;26(3):319–33. doi: 10.1111/bpa.12298 (PMC4950048; doi:10.1111/bpa.12298)

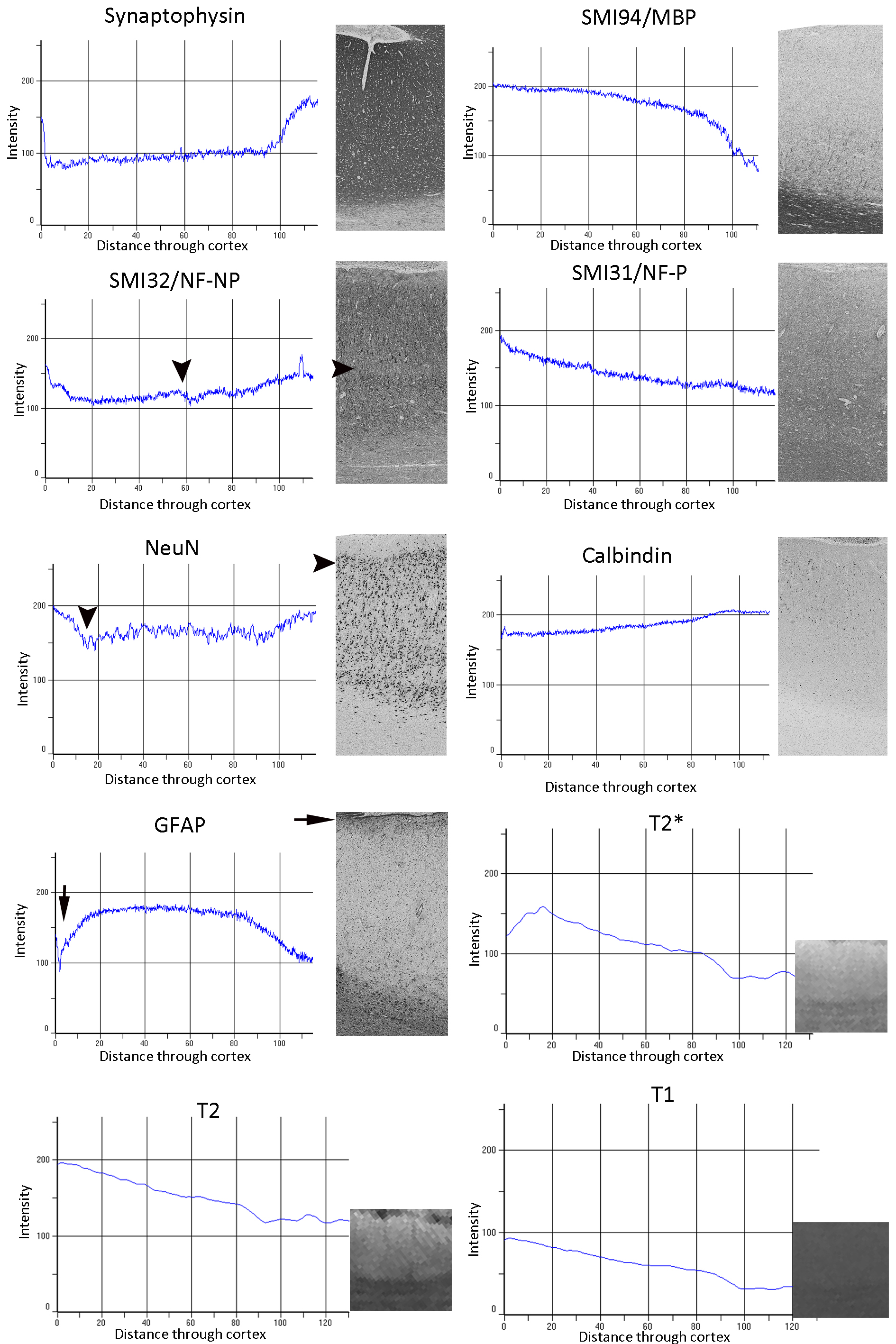

Supplement: Supplementary file 1 — Figure S1. Line profile analysis (LPA) for case 6 (no cortical dyslamination). Figure S2. Line profile analysis (LPA) for case 9 (no cortical dyslamination). Figure S3. Line profile analysis (LPA) for case 11 (post‐mortem sample with no dyslamination). Figure S4. (A) Line profile analysis (LPA) in FCD IIA (case 4). Figure S5. Line profile analysis (LPA) for case 10 in FCD IIB. [file BPA-26-319-s001.zip › BPA_12298_Supp0001_FS1.jpg]

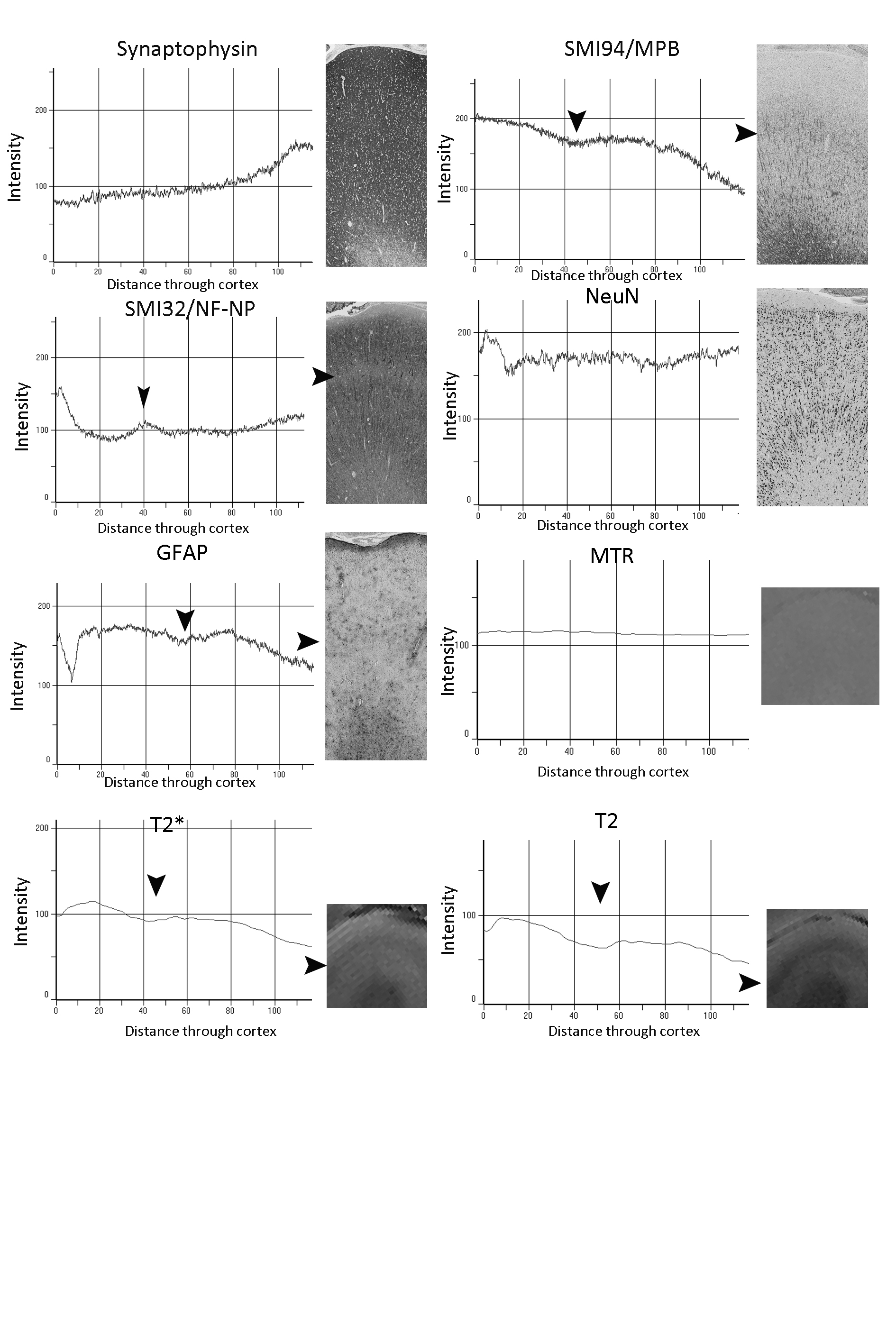

Supplement: Supplementary file 1 — Figure S1. Line profile analysis (LPA) for case 6 (no cortical dyslamination). Figure S2. Line profile analysis (LPA) for case 9 (no cortical dyslamination). Figure S3. Line profile analysis (LPA) for case 11 (post‐mortem sample with no dyslamination). Figure S4. (A) Line profile analysis (LPA) in FCD IIA (case 4). Figure S5. Line profile analysis (LPA) for case 10 in FCD IIB. [file BPA-26-319-s001.zip › BPA_12298_Supp0002_FS2.jpg]

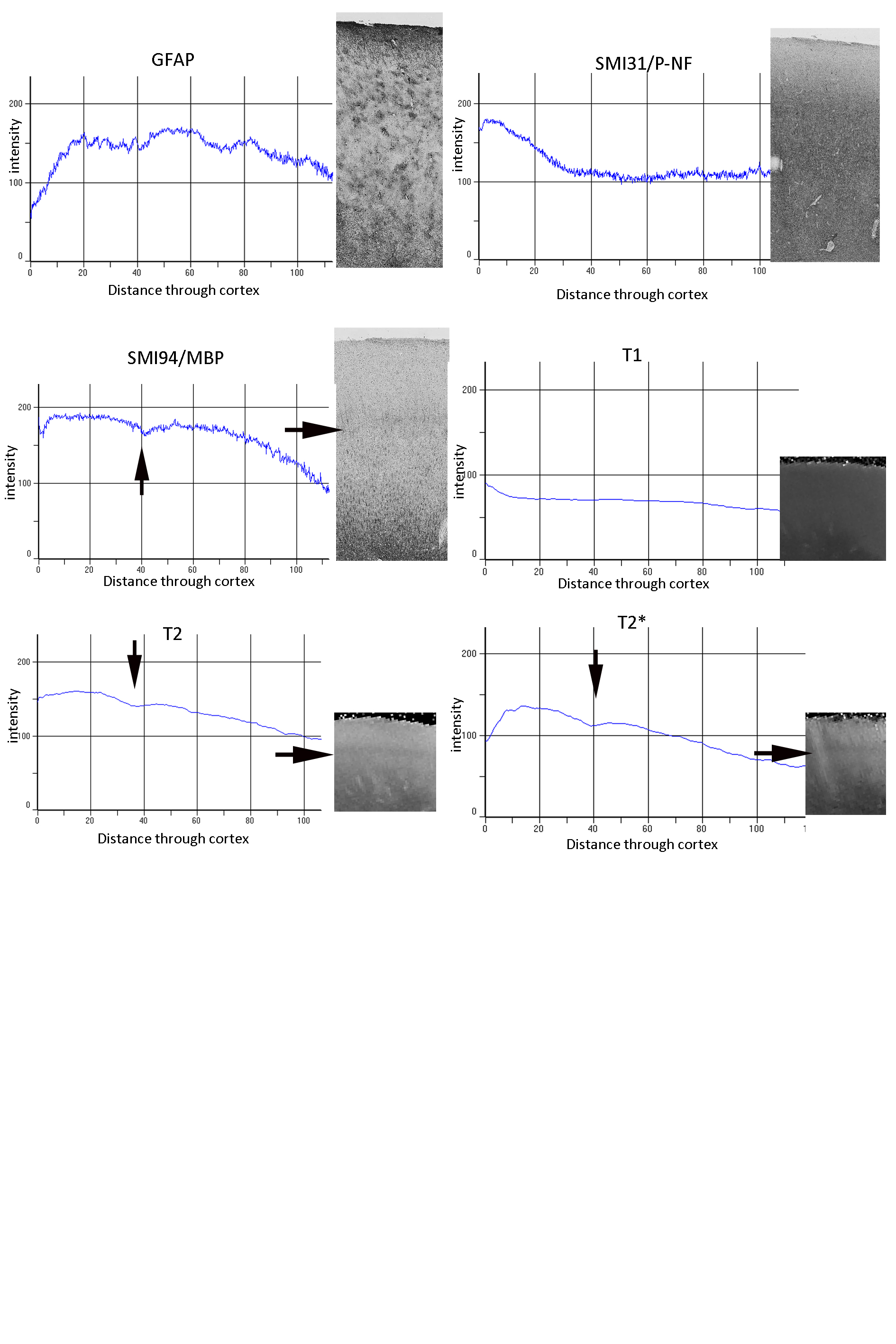

Supplement: Supplementary file 1 — Figure S1. Line profile analysis (LPA) for case 6 (no cortical dyslamination). Figure S2. Line profile analysis (LPA) for case 9 (no cortical dyslamination). Figure S3. Line profile analysis (LPA) for case 11 (post‐mortem sample with no dyslamination). Figure S4. (A) Line profile analysis (LPA) in FCD IIA (case 4). Figure S5. Line profile analysis (LPA) for case 10 in FCD IIB. [file BPA-26-319-s001.zip › BPA_12298_Supp0003_FS3.jpg]

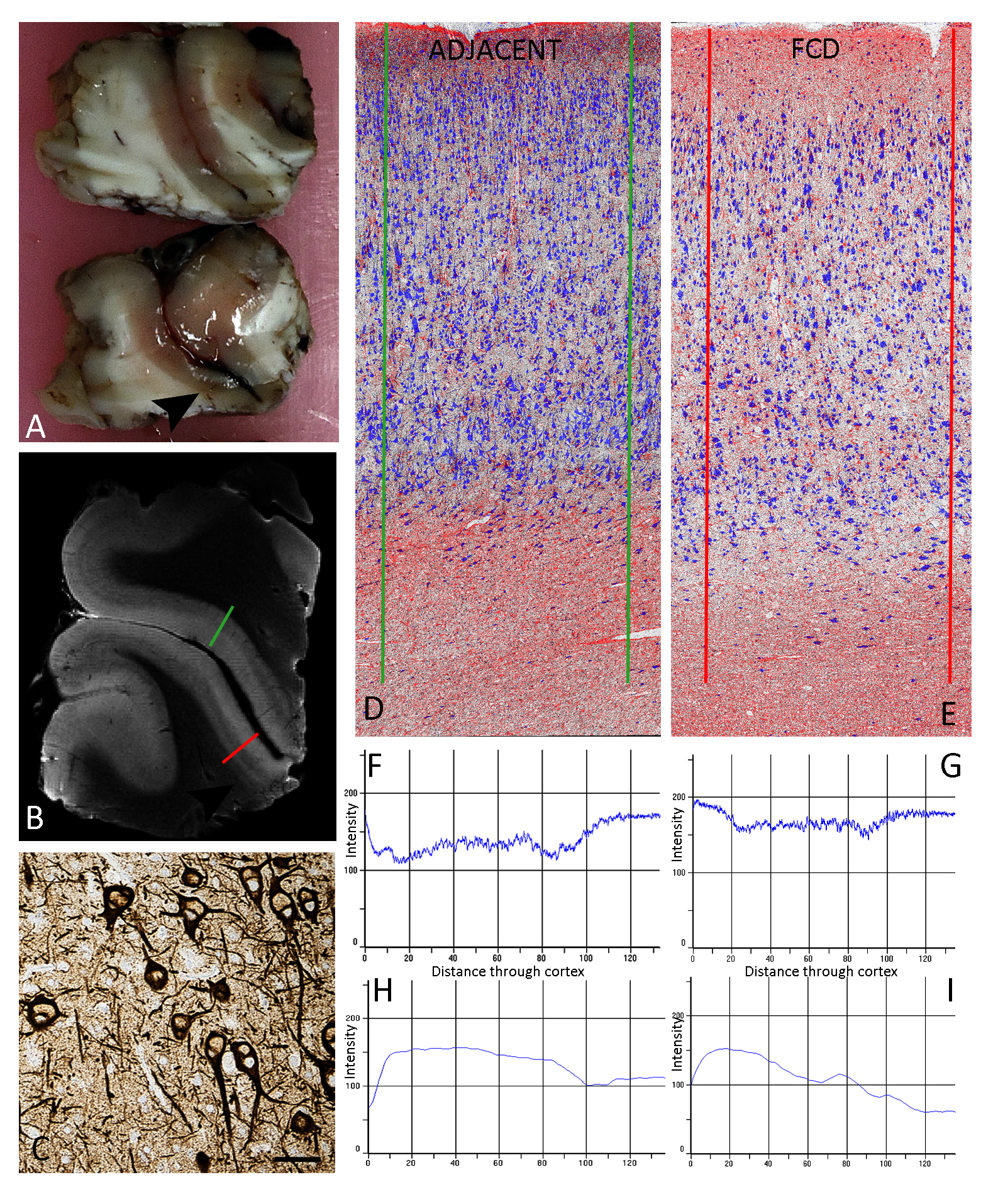

Supplement: Supplementary file 1 — Figure S1. Line profile analysis (LPA) for case 6 (no cortical dyslamination). Figure S2. Line profile analysis (LPA) for case 9 (no cortical dyslamination). Figure S3. Line profile analysis (LPA) for case 11 (post‐mortem sample with no dyslamination). Figure S4. (A) Line profile analysis (LPA) in FCD IIA (case 4). Figure S5. Line profile analysis (LPA) for case 10 in FCD IIB. [file BPA-26-319-s001.zip › BPA_12298_Supp0004_FS4a.tif]

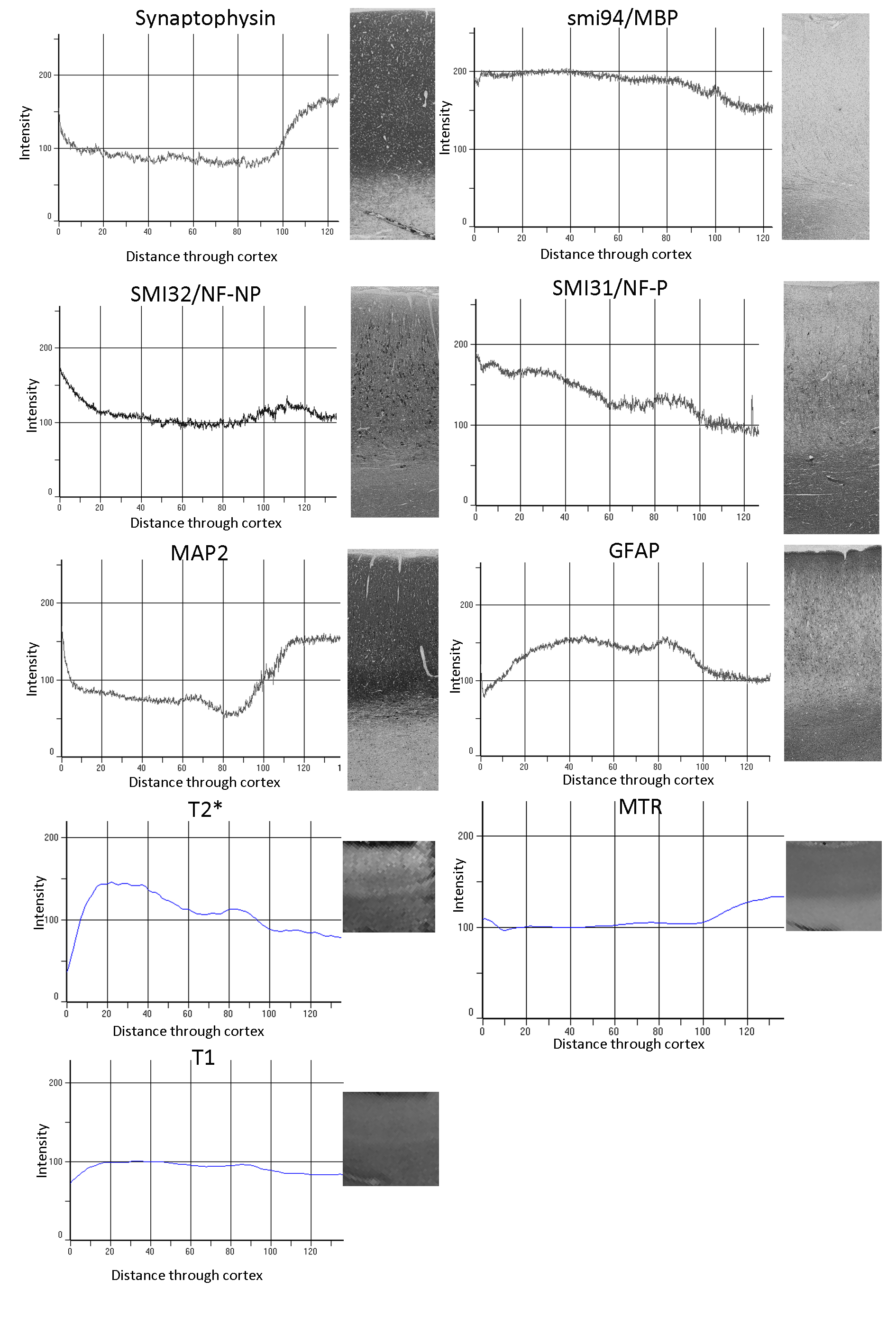

Supplement: Supplementary file 1 — Figure S1. Line profile analysis (LPA) for case 6 (no cortical dyslamination). Figure S2. Line profile analysis (LPA) for case 9 (no cortical dyslamination). Figure S3. Line profile analysis (LPA) for case 11 (post‐mortem sample with no dyslamination). Figure S4. (A) Line profile analysis (LPA) in FCD IIA (case 4). Figure S5. Line profile analysis (LPA) for case 10 in FCD IIB. [file BPA-26-319-s001.zip › BPA_12298_Supp0004_FS4b.jpg]

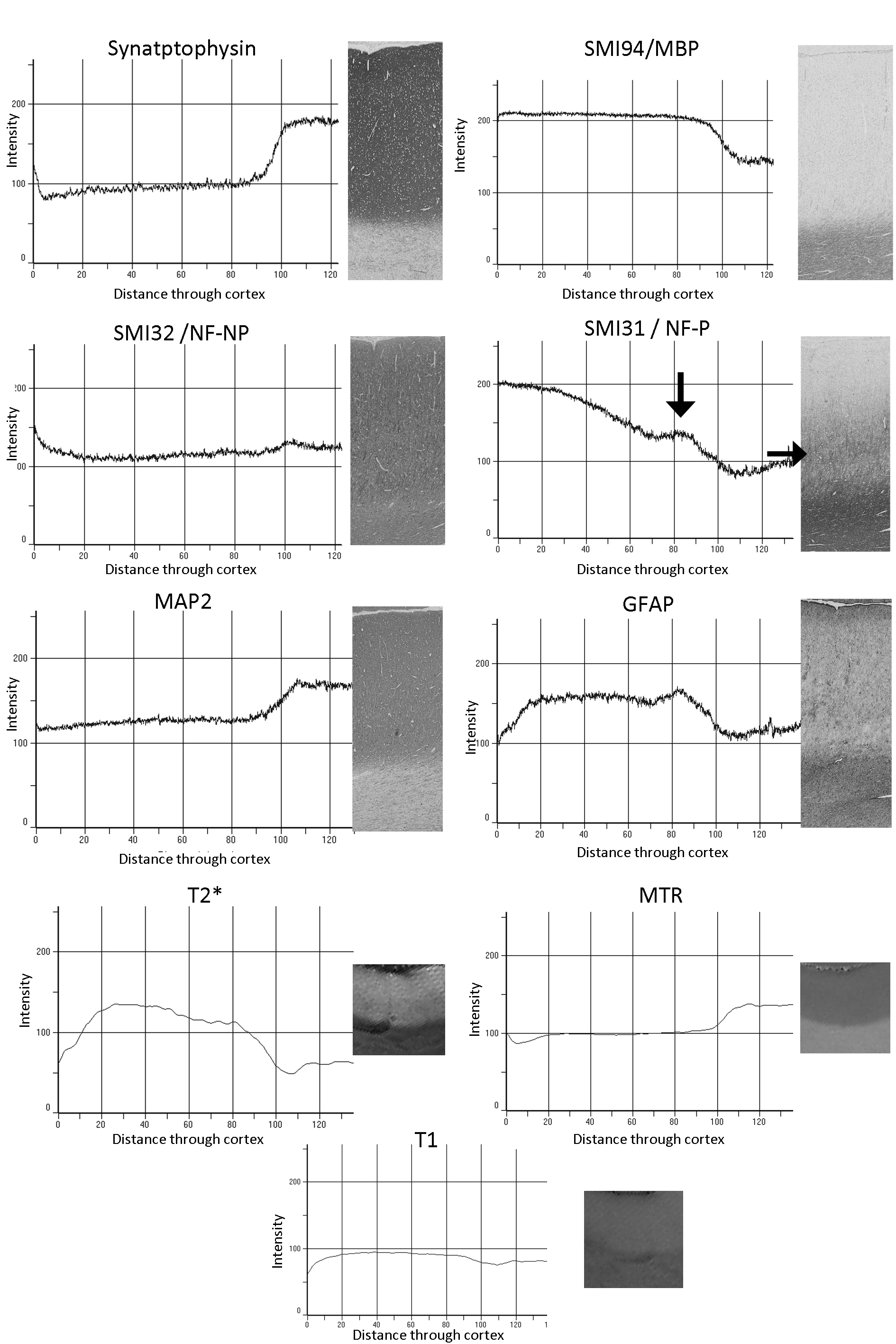

Supplement: Supplementary file 1 — Figure S1. Line profile analysis (LPA) for case 6 (no cortical dyslamination). Figure S2. Line profile analysis (LPA) for case 9 (no cortical dyslamination). Figure S3. Line profile analysis (LPA) for case 11 (post‐mortem sample with no dyslamination). Figure S4. (A) Line profile analysis (LPA) in FCD IIA (case 4). Figure S5. Line profile analysis (LPA) for case 10 in FCD IIB. [file BPA-26-319-s001.zip › BPA_12298_Supp0004_FS4c.jpg]

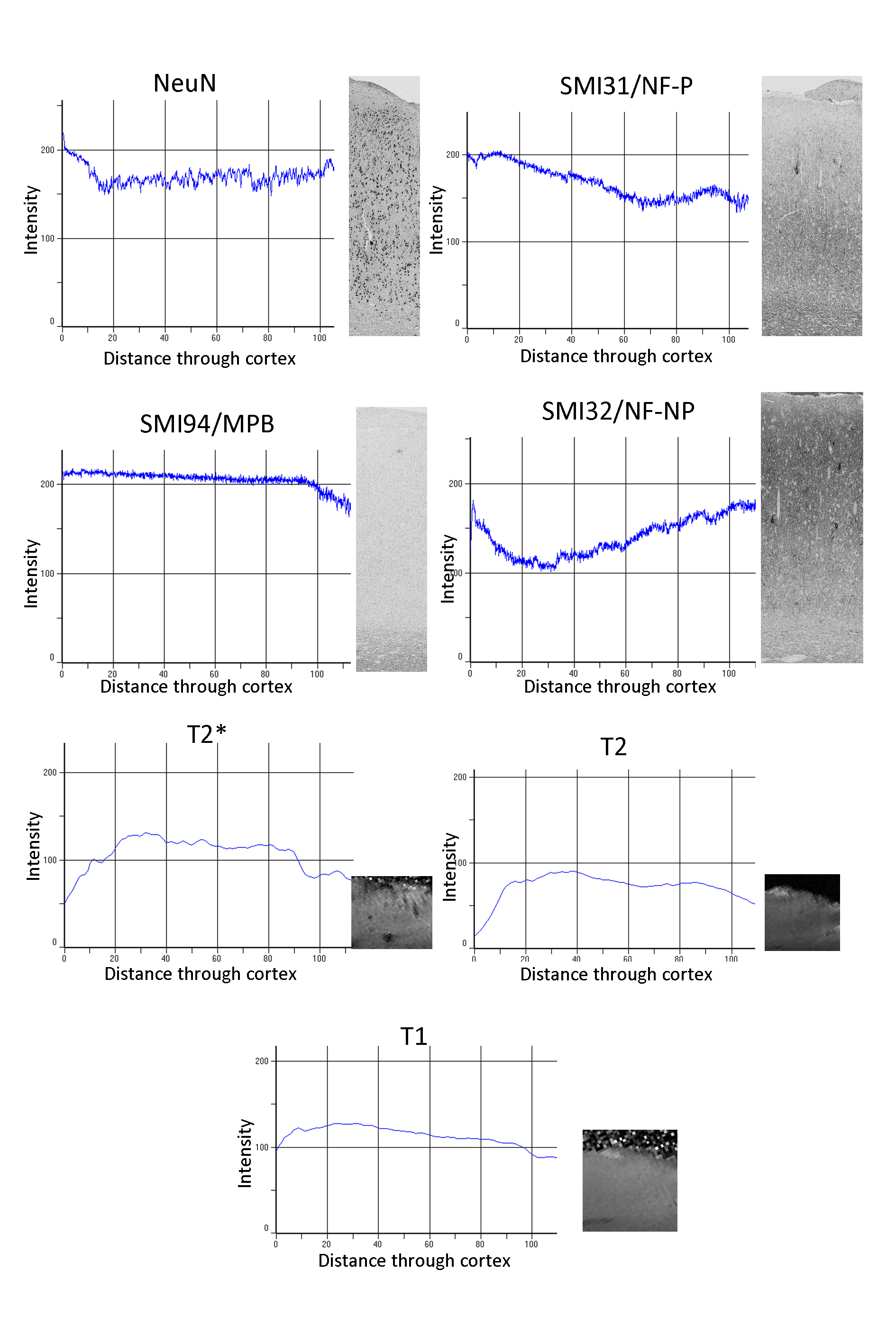

Supplement: Supplementary file 1 — Figure S1. Line profile analysis (LPA) for case 6 (no cortical dyslamination). Figure S2. Line profile analysis (LPA) for case 9 (no cortical dyslamination). Figure S3. Line profile analysis (LPA) for case 11 (post‐mortem sample with no dyslamination). Figure S4. (A) Line profile analysis (LPA) in FCD IIA (case 4). Figure S5. Line profile analysis (LPA) for case 10 in FCD IIB. [file BPA-26-319-s001.zip › BPA_12298_Supp0005_FS5.jpg]
